# Molecular and biological characterization of a partitivirus from *Paecilomyces variotii*

**DOI:** 10.1099/jgv.0.001925

**Published:** 2023-11-28

**Authors:** Sidra Hassan, Urayama Syun-ichi, Saba Shabeer, Chien-Fu Wu, Hiromitsu Moriyama, Robert H. A. Coutts, Ioly Kotta-Loizou, Atif Jamal

**Affiliations:** ^1^​ Department of Plant and Environmental Protection, PARC Institute of Advanced Studies in Agriculture (Affiliated with Quaid-i-Azam University), National Agricultural Research Centre, Islamabad, 45500, Pakistan; ^2^​ Laboratory of Fungal Interaction and Molecular Biology (donated by IFO), Department of Life and Environmental Sciences, University of Tsukuba, 1-1-1 Tennodai, Tsukuba, Ibaraki 305-8577, Japan; ^3^​ Department of Bioscience, COMSATS University, Islamabad, 44000, Pakistan; ^4^​ Crop Diseases Research Institute (CDRI), National Agricultural Research Centre, Park Road, Islamabad, 45500, Pakistan; ^5^​ Laboratory of Molecular and Cellular Biology, Department of Applied Biological Sciences, Tokyo University of Agriculture and Technology, 3-5-8, Saiwaicho, Fuchu, Tokyo 184-8509, Japan; ^6^​ Department of Clinical, Pharmaceutical and Biological Science, School of Life and Medical Sciences, University of Hertfordshire, Hatfield, AL10 9AB, UK; ^7^​ Department of Life Sciences, Faculty of Natural Sciences, Imperial College London, London, SW7 2AZ, UK

**Keywords:** *Paecilomyces variotii*, double-stranded RNA, *Partitiviridae*, zetapartitivirus, biological control, growth, metabolism

## Abstract

*Paeciliomyces variotii* is a thermo-tolerant, ubiquitous fungus commonly found in food products, indoor environments, soil and clinical samples. It is a well-known biocontrol agent used against phytopathogenic fungi and its metabolites have many industrial applications. Rare reports of *P. variotii-*related human infections have been found in the medical literature. In this study, we report for the first time the infection of *P. variotii* isolated from a soil sample collected in a rice field with a double-stranded RNA virus, Paeciliomyces variotii partitivirus 1 (PvPV-1) in the family *Partitiviridae. P. variotii* harboured icosahedral virus particles 30 nm in diameter with two dsRNA segments 1758 and 1356 bp long. Both dsRNA1 and dsRNA2 have a single open reading frame encoding proteins of 63 and 40 kDa, respectively. These proteins have significant similarity to the RNA-dependent RNA polymerase and capsid protein encoded by the genomic segments of several viruses from the family *Partitiviridae*. Phylogenetic analysis revealed that PvPV-1 belongs to the family *Partitiviridae* but in an unclassified group/genus, tentatively nominated Zetapartitivirus. PvPV-1 was found to increase the growth rate of the host fungus, as indicated by time course experiments performed on a range of different media for virus-infected and virus-free isogenic lines. Further, dual-culture assays performed for both isogenic lines confirmed the antagonistic potential of *P. variotii* against other phytopathogenic fungi. The findings of this study assist us in understanding *P. variotii* as a potential biocontrol agent, together with plant–fungus–virus interactions.

## Introduction

The soil hosts a highly diverse fungal network that is actively involved in the disintegration of organic matter, thus shaping the structures and functions of microbial and plant communities. Soil fungi have roles in organic matter stabilization by producing enzymes fixing nitrogen, controlling root pathogens and protecting against drought [1–4]. The diversity of fungal communities is greatly influenced by plant diversity and reciprocally fungal communities affect plant growth through different associations, including mutualism, antagonism or pathogenicity [5].

All life forms are infected by viruses and fungi are no exception. Mycoviruses or fungal viruses are found in plant-associated fungi, but also in saprophytes and wild mushrooms [6–8]. Mycoviruses are currently grouped into 27 families (ICTV Master Species List 2022 MSL38.v3; https://ictv.global/msl), comprising linear double-stranded (ds) RNA, linear positive-sense or negative-sense, single-stranded (ss) RNA, and circular ss DNA genomes [9–13]. These viruses infect different genera of fungi, including *Alternaria*, *Aspergillus*, *Botrytis*, *Fusarium*, *Mortierella*, *Mucor*, *Penicillium*, *Rhizopus*, *Sclerotinia* and *Trichoderma* [14, 15]. However, the occurrence of mycoviruses in the genus *Paecilomyces* (family *Thermoascaceae*, order Eurotiales, class Eurotiomycetes, phylum Ascomycota) has not been studied in depth. Mycovirus infection was previously reported for a few species in *Paecilomyces*, including *P. fumosoroseus* (later *Isaria fumosorosea*), *P. farinosus* (later *Isaria farinosa*)*, P. amoenoroseus* and *P. lilacinus* (later *Purpureocillium lilacinum*) [16, 17].

*Paeciliomyces variotii* has a potential role in controlling pests and diseases and this ability makes it an eco-friendly alternative to other conventional agricultural practices. *P. variotii* has been shown to be an effective antagonist, reducing disease severity against phytopathogenic fungi and bacteria, including *Podosphaera xanthii*, *Fusarium oxysporum*, *Macrophomina phaseolina*, *Mycosphaerella melonis* and *Xanthomonas* spp. [18]. *P. variotii* was found to be compatible with fungicides such as cymoxanil, mancozeb, triadimenol, copper hydroxide and copper oxychloride [18]. Therefore, *P. variotii* may be utilized as a biological control agent against several diseases and should be considered for potential integration into advanced pest management strategies.

*P. variotii* can also produce secondary metabolites, including the antibiotic ascofuranone, peptides, polyketides, naphthopyranones, sphingofungins, novel branched fatty acids, eicosenoic acids, anacardic acid analogues and high-value volatile compounds. Many of these products play a role in improving animal and human health or as herbicidal agents for agrochemical markets [19].

The presence of a mycovirus in *P. variotii* may result in virus–host interactions influencing *P. variotii* as a biocontrol agent, by affecting growth and metabolite production. At present, the focus of mycovirology is expanding from plant pathogenic fungi and mycovirus-mediated hypovirulence to other interactions; effects such as hypervirulence, metabolite production, drug resistance, control of endophytic traits and other mycovirus-facilitated phenotypes are also central to current research [20]. For fungi such as *P. variotii,* which act as natural biocontrol agents for other phytopathogenic fungi [18], investigations of virulence within this three-way interaction are paramount. This investigation is the first to report the presence and genomic sequence of a partitivirus in *P. variotii* and assess the effects of mycovirus infection on the biology of the host fungus.

## Methods

### Isolation and identification of *P. variotii*

*P. variotii* was isolated from soil samples collected in rice fields from different regions of Khyber Pakhtunkhwa, Pakistan. Soil samples were serially diluted, inoculated on potato dextrose agar (PDA, Millipore) and incubated at 25 °C for 5–7 days followed by single colony isolation on PDA. Total nucleic acid and dsRNA extractions were performed [21, 22], and fungal mycelia were stored in 40 % glycerol at −20 °C for future use. For molecular identification of fungi, the internal transcribed spacer (ITS) regions of nuclear DNA were amplified by polymerase chain reaction (PCR) using ITS1 and ITS4 primers [23, 24].

### Fragmented and primer-ligated dsRNA sequencing (FLDS)

Purified dsRNA was subjected to a novel method of library construction called FLDS [22]. This method consists of physical fragmentation of dsRNA following cellulose column chromatography, synthesis of complementary (c) DNA by reverse transcription (RT) using a modified rapid amplification of cDNA ends (RACE) method, and library construction and amplification of cDNA via PCR. Finally, dsRNA sequencing was performed using the KAPA Hyper Prep kit Illumina Platform (Kapa Biosystems, Woburn MA, USA). Illumina NovaSeq 6000/PE150 was used to determine 150 bp of the paired end sequence of each fragment. Raw sequencing data were processed through the FLDS pipeline (available in GitHub). CLC Genomic Workbench version 11.0 was used for *de novo* assembly of contigs >300 nt (other options were default). Full-length recovery of dsRNA segments was confirmed by manual analysis using CLC Genomic Workbench version 11.0, Genetyx version 14 and Tablet viewer version 1.19.09.03.

### Multiple sequence alignment, phylogenetic analysis and secondary structure prediction

The online Multiple Alignment using Fast Fourier Transform (MAFFT v7.511) tool was used for multiple sequence analysis [25]. Molecular Evolutionary Genetics Analysis (MEGA) 11 software was used for phylogenetic analysis [26]. The secondary structures of the 5′- and 3′-termini of dsRNAs were determined using the online RNAfold (2.5.1) tool [27].

### Purification of virus-like particles (VLPs)

VLPs were purified as described elsewhere [28] with some modifications. Fungal mycelium (50 g) was homogenized for ~3 min in two volumes of TE buffer (50 mM Tris–Cl pH 7.5, 1 mM EDTA pH 8.0) in a blender. The homogenate was filtered through Miracloth and put into 250 ml Nalgene bottles. Centrifugation was performed at 10 000 ***g*** for 20 min. The VLPs in the supernatant were then precipitated with 10 % (w/v) polyethylene glycol (PEG)-6000 and 0.6 M NaCl by stirring at 4 °C overnight. Centrifugation for 20 min at 10 000 ***g*** was performed, and the pellet containing the VLPs was resuspended in TE buffer and recentrifuged at 10000 ***g*** for 20 min. The collected supernatant containing the VLPs was then subjected to ultracentrifugation at 105 000 ***g*** for 90 min. The pellet containing the VLPs was resuspended in 1 ml TE buffer and transferred to 1.5 ml Eppendorf tubes. The tubes were again centrifuged at 10 000 ***g*** for 20 min to further clarify the supernatant containing the VLPs. Virus dsRNA was extracted with phenol–chloroform–isoamyl alcohol, electrophoresed on 1 % (w/v) agarose gel and visualized under ultraviolet light.

### Transmission electron microscopy (TEM) and sodium dodecyl sulfate polyacrylamide gel electrophoresis (SDS-PAGE)

Purified VLPs were visualized using TEM. Carbon Film 300 Mesh copper grids were glow discharged for 1 min under vacuum and 3 µl of sample was added to the surface of the grids and incubated for 1 min. Excess sample was wiped from the grid manually. Uranyl acetate (2 %) staining solution was added to the surface of the grid and incubated for 1 min. Excess stain was again blotted off the grid manually and the grids were observed by TEM operating at 120 kV using a LAB6 filament. Emission was set to 4 μA, and images were recorded at magnifications ranging from 46 000–67 000×, with an exposure time of 1.4 s. SDS-PAGE was performed for the purified VLPs [29], followed by SYPRO-RUBY (Thermo Fisher Scientific) staining.

### Curing of mycovirus infection with cycloheximide treatment

Fungal isolates were cured of mycovirus infection by treatment with cycloheximide [30–32], which was filter-sterilized and added into PDA. Growth was restricted by high concentrations of cycloheximide, therefore the culture was selected and placed into a 2 ml Eppendorf tube containing water. Following shaking and dilution, the contents of the tube were inoculated on water agar plates. Individual spores were selected and inoculated onto freshly prepared PDA plates and subsequently potato dextrose broth (PDB) for nucleic acid extraction. Virus-free isogenic lines were confirmed by performing RT-PCR amplification using a standard protocol with purified dsRNA (9 µl) as template, which was heat-denatured with 100 % dimethyl sulfoxide (DMSO; 40 µl) and random hexamers (1 µl) at 65 °C for 20 min. The denatured dsRNA was snap-cooled on ice and precipitated using 0.1 vol of 3 M sodium acetate (0.5 µl) pH 5.2 and 2.5 volumes of absolute ethanol. The mixture was incubated at −80 °C for 30 min or −20 °C overnight followed by centrifugation at 21 380 ***g*** for 20 min. The pellet was washed with 70 % ethanol, air-dried, incubated for 10 min on ice and resuspended in a first-strand cDNA synthesis reaction [4 µl nuclease-free water, 4 µl RT buffer (5×), 5 µl deoxyribonucleotides (dNTPs; 2 mM), 2 µl dithiothreitol (DTT; 0.1 M), 3 µl random hexamer primers (10 pmol)], ribonuclease inhibitor (Thermo Fisher Scientific) and RTase (SuperScript, Thermo Scientific). Next, the reaction mixture was incubated at 37 °C for 90 min. The cDNA was diluted 1 : 4 (10 µl of cDNA and 40 µl of sterile distilled water) and PCR was performed using a virus-specific reverse primer (5′-GAT AGA GCG CCA CGC ACA CCG-3′) and forward primer (5′-CCG TAC AAA CGA GGA GTG TTG-3′), designed from the virus sequences obtained through FLDS to amplify a specific fragment of the virus genome. The provenance of the RT-PCR amplicon was confirmed by restriction endonuclease digestion with *Eco*RV (Promega) according to the manufacturer’s instructions.

### Horizontal transfer of the mycovirus via hyphal fusion

The mycovirus was transferred from the original virus-infected isolate (donor) to one cured of infection with cycloheximide treatment (recipient). The two isolates were allowed to grow and fuse on PDA, followed by selection of different regions from both donor and recipient, and sub-culturing on fresh PDA plates. The cultures were subsequently inoculated in PDB, and their infection status was determined by dsRNA extraction and agarose gel electrophoresis.

### Radial expansion, biomass production and metabolic assays

For radial expansion assays and assessment of colony morphology, equal numbers of spores of virus-infected and virus-free isogenic lines were centrally inoculated on PDA and incubated at 28 °C. Other media investigated included Sabouraud dextrose agar (SDA), yeast extract and sucrose agar (YES) and Czapek Dox agar (CDA) complete and minimal media. Colony radii were measured and recorded every 24 h over a period of 4 days until the colonies completely covered the plates.

For biomass production assays, equal numbers of spores of virus-infected and virus-free isogenic lines were inoculated into 10 ml PDB in 50 ml Falcon tubes, incubated at 28 °C and further processed once visible mycelial mats had grown. Individual cultures were filtered through Whatman paper, wet mycelial weights were recorded, mycelia were wrapped in filter paper and dried for 24 h, and dry mycelial weights were recorded.

2,3-bis (2-methoxy-4-nitro-5-sulfophenyl)−5-carboxanilide-2H-tetrazolium (XTT) metabolic assays were performed as described elsewhere [33, 34]. Fungi were grown on PDA until sporulation occurred and conidia were collected in 1× phosphate-buffered saline (PBS) and counted using a haemocytometer. Fungal conidia (10^7^ spores) were seeded in malt extract broth (MEB), YES and different formulations of CD liquid minimal media in 96-well plates and allowed to grow at 25 °C for 24 h. XTT–menadione–liquid media solution was added to each well and the 96-well plates were incubated in the dark for 1 h. The supernatants from each well were transferred to a fresh 96-well plate and assayed using a plate reader at 490 nm. All experiments were performed in triplicate and the results were analysed statistically using the two-tailed Student’s *t*-test or two-way analysis of variance (ANOVA) in GraphPad Prism.

### Dual-culture assays

The antagonistic potential of *P. variotii* virus-infected and virus-free isogenic lines was evaluated against other fungi, including *Aspergillus terreus*, *Hypoxylon* spp. and *Penicillium citrinum* by performing dual-culture experiments [35, 36]. These fungal isolates were isolated from tobacco field soil samples and tested negative for mycovirus infection following dsRNA extraction. Equal numbers of spores of both *P. variotii* and the antagonist were inoculated on freshly prepared PDA media, 2 cm away from the edge of the PDA plates. In the control group, PDA plates were inoculated solely with the antagonist in the absence of *P. variotii*. Plates were kept in an incubator at 25 °C for 5–7 days until inhibition could be observed. All experiments were performed in octuplicate and the following formula was used to calculate % inhibition.

% inhibition = (*A*−*B*)/*A*×100

*A*=colony diameter of fungal pathogen in the control group

*B*=colony diameter of fungal pathogen in the treated group

## Results

### Identification of a dsRNA mycovirus in a soil isolate of *P. variotii*

**Fig. 1.**
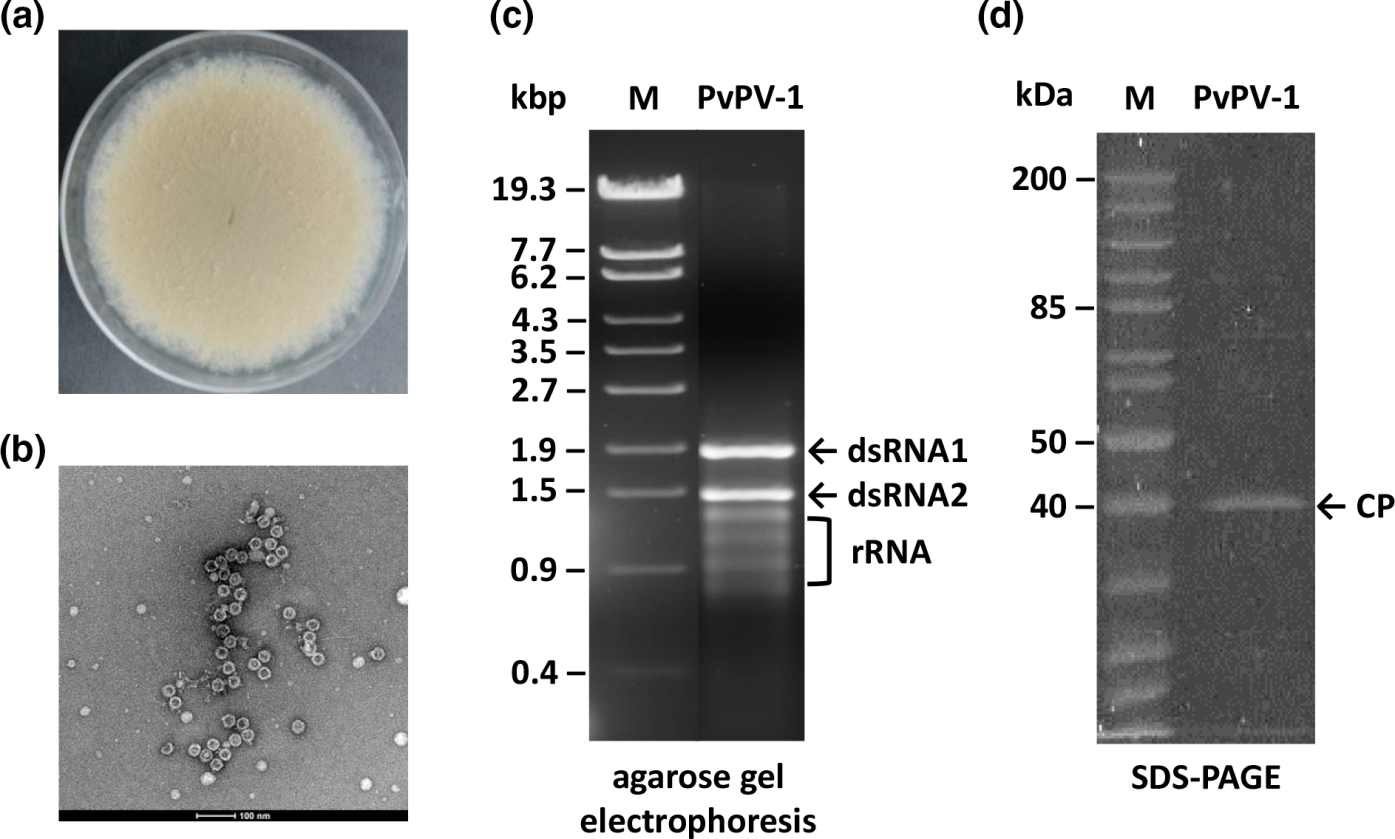
PvPV-1 components. (**a**) Colony morphology of *P. variotii* isolate 15R on PDA. (**b**) TEM visualization of icosahedral VLPs 30 nm in diameter extracted from *P. variotii*. (**c**) Electrophoretic profile of the dsRNA elements extracted from *P. variotii* on a 1 % (w/v) agarose gel, showing two dsRNA bands approximately 1.5 and 1.9 kbp in size. The molecular sizes of λ-EcoT141-digested DNA markers are indicated on the left of the gel. (**d**) Electrophoretic profile of the VLPs on 10 % (w/v) SDS-PAGE analysis of the purified virus particles showing a CP approximately 40 kDa in mass. The molecular sizes of PAGE Ruler unstained protein ladders are indicated on the left of the gel.

A total of 8 rice field soil samples were screened, resulting in 46 fungal isolate representatives of genera such as *Aspergillus*, *Fusarium*, *Hypoxylon* and *Trichoderma*. Following screening for mycovirus infection, five tested positive for mycoviral infection, including a *P. variotii* isolate found to be infected with dsRNA elements, potentially representing the genome or the replicative form of an RNA mycovirus. One fungal isolate, 15R, was found to harbour such dsRNA elements between 1 and 2 kbp in size. The colony morphology of the virus-infected *P. variotii* 15R isolate and its dsRNA electrophoretic profile are shown in Fig. 1a, c, respectively. Minor bands observed in Fig. 1c are potentially ribosomal (r) RNAs from the fungal host, since no S1 nuclease treatment was performed following cellulose column chromatography. FLDS was performed using this extract, and only one virus whose genome corresponds to the two clear dsRNA bands was found in the *P. variotii* 15R isolate. Following purification of VLPs and visualization by TEM, icosahedral VLPs 30 nm in diameter (Fig. 1b) were observed. The proteinaceous component of the VLPs was subjected to SDS-PAGE and staining with SYPRO-RUBY, revealing a single band approximately 40 kDa in size (Fig. 1d).

### Molecular characterization of a novel partitivirus infecting *P. variotii*

The purified dsRNA elements were subjected to FLDS for complete sequence determination. Two contigs containing 57 and 37 % of assembled reads were determined as major (>2.5 % of assembled reads) contigs. Based on the mapping analysis, these two contigs were confirmed to be complete sequences of two dsRNAs, 1758 and 1356 bp in size. The complete nucleotide sequences of dsRNA 1 and dsRNA 2 were submitted to DNA Data Bank of Japan (DDBJ) with accession numbers LC764452 and LC764453, respectively.

**Fig. 2.**
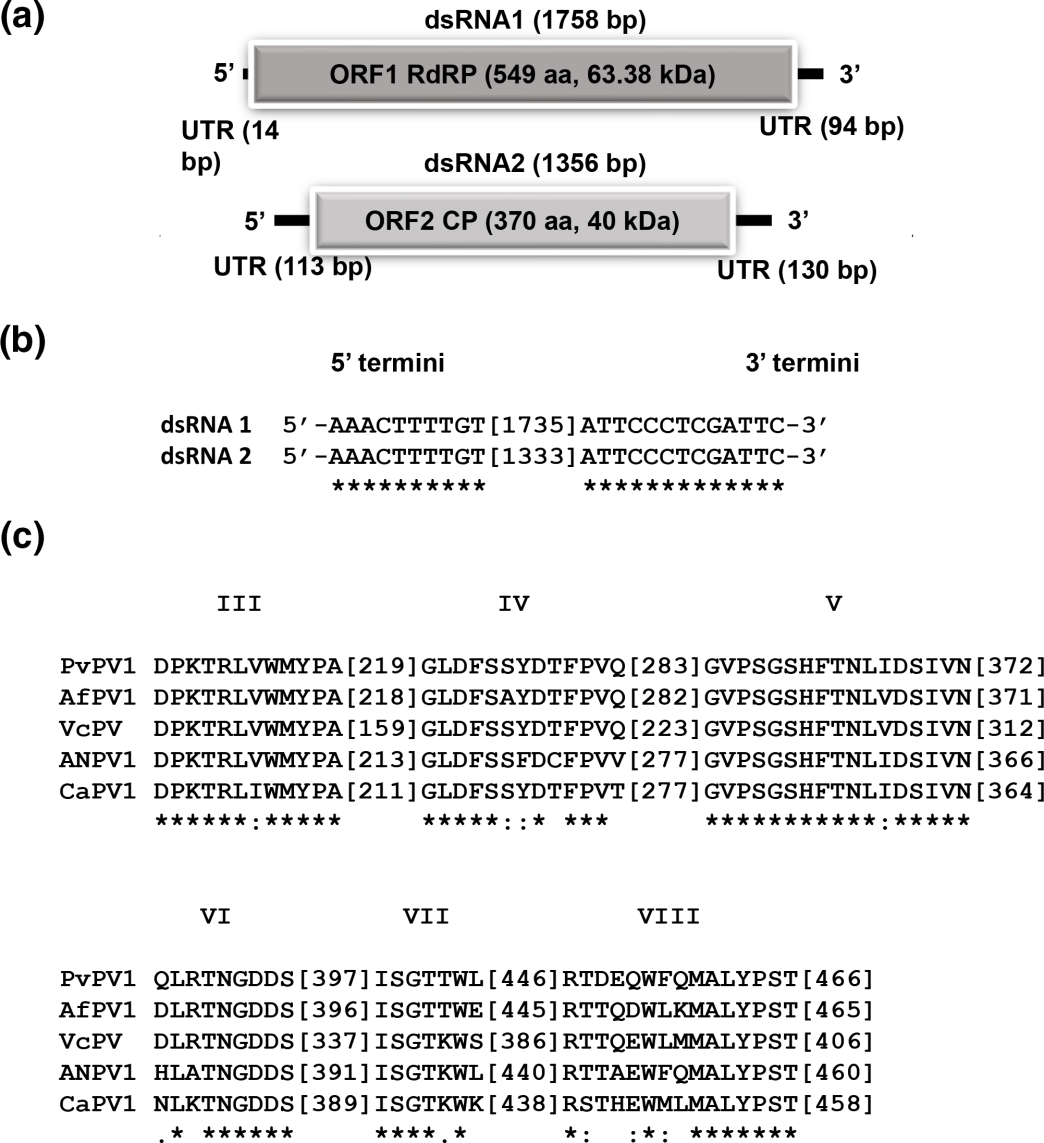
PvPV-1 genomic organization. (**a**) Schematic representation of PvPV-1 dsRNA 1 and dsRNA 2. Each dsRNA is shown as a black line and each ORF as a grey box. (**b**) Nucleotide sequence alignment of the 5′- and 3′-termini of the coding strands of the two PvPV-1 dsRNAs using MAFFT. Asterisks signify identical nucleotides. (**c**) Amino acid sequence alignment of the core RdRP motifs (III–VIII) of PvPV-1 and selected members in the family *Partitiviridae* using MAFFT. Asterisks signify identical residues; colons signify highly conserved residues and single dots signify less conserved but related residues.

Sequence analysis revealed that both dsRNA1 and dsRNA2 have a single open reading frame (ORF) encoding proteins 63 and 40 kDa in size, respectively (Fig. 2a). The Basic Local Alignment Sequence Tool for proteins (BLASTp) used to search public databases revealed that the dsRNA1 ORF encodes a protein that shares clear similarities with the RNA-dependent RNA polymerase (RdRP) of several other viruses in the family *Partitiviridae,* including Valsa cypri partitivirus (84 % identity), Botryosphaeria dothidea virus 1 (83 % identity), Aspergillus flavus partitivirus 1 (82 % identity), Aspergillus niger partitivirus 1 (80 % identity) and Colletotrichum acutatum RNA virus 1 (78 % identity) (Table S1). Multiple alignment of the putative RdRP amino acid (aa) sequence and other related sequences from the family *Partitiviridae* was performed, revealing the presence of all six conserved RdRP motifs [37] (Fig. 2c). Similarity between the putative protein encoded by the dsRNA2 ORF and Partitiviridae capsid protein (CP) sequences were found for Botryosphaeria dothidea virus 1 (60 % identity), Aspergillus niger partitivirus 1 (58 % identity), Aspergillus flavus partitivirus 1 (57 % identity), Colletotrichum acutatum RNA virus 1 (57 % identity) and Valsa cypri partitivirus (56 % identity) (Table S1). Therefore, the novel virus was named Paecilomyces variotii partitivirus 1 (PvPV-1).

**Fig. 3.**
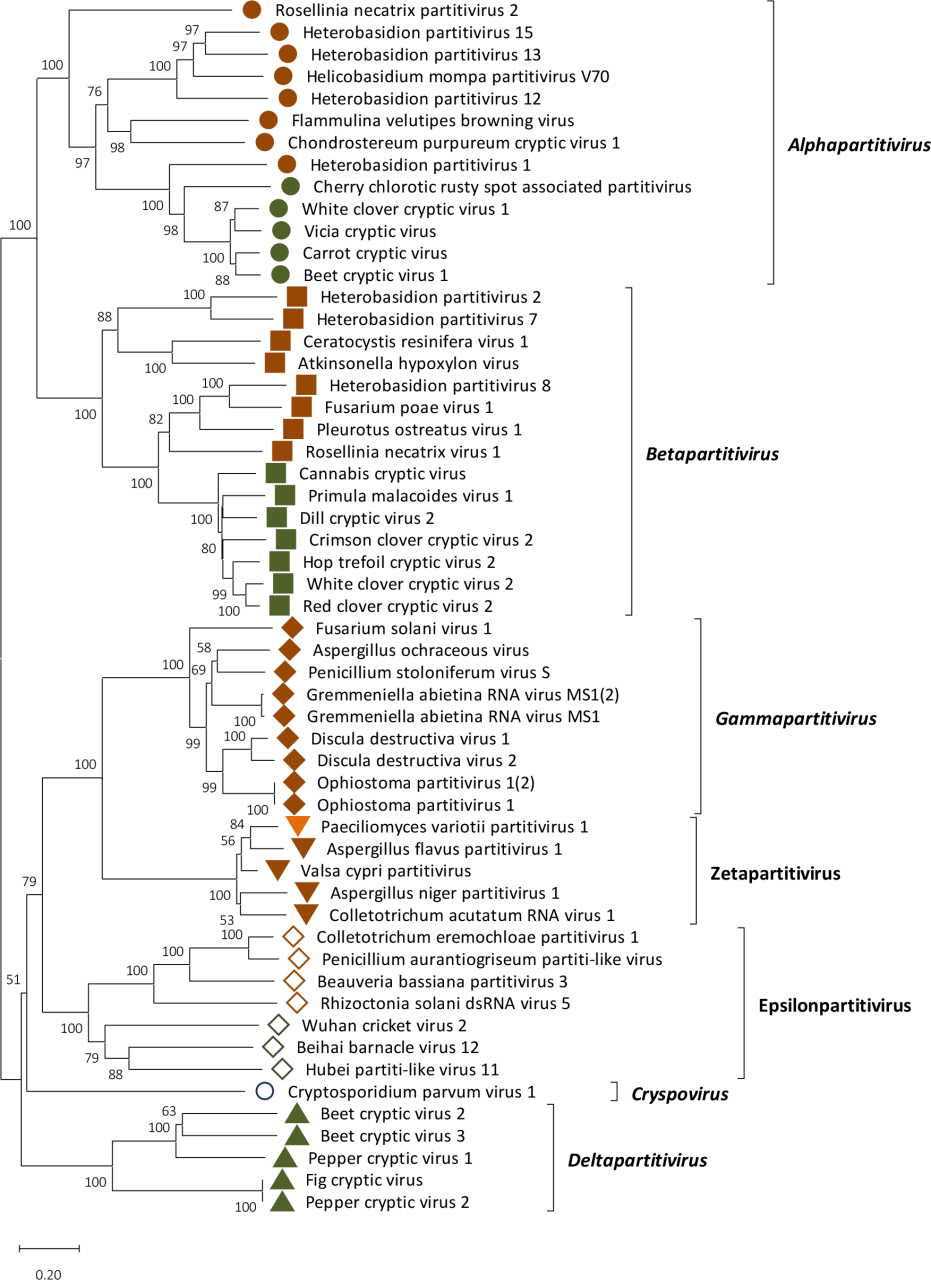
PvPV-1 phylogeny. Phylogenetic analysis of PvPV-1 and selected members of the family *Partitiviridae* based on their RdRP amino acid sequences. A multiple alignment of RdRP amino acid sequences was produced using muscle as implemented using mega 11. A neighbour-joining (NJ) phylogenetic tree was constructed using MEGA 11. Bootstrap percentages (1000 replicates) over 50 % are shown. Tips labelled with brown, green and blue shapes indicate that the virus host is respectively fungal, plant or protozoon. The orange rhombus indicates the position of PvPV-1.

PvPV-1 phylogeny was examined by constructing a phylogenetic tree using RdRP aa sequences from viruses in the family *Partitiviridae* (Fig. 3). This analysis revealed that PvPV-1 and related viruses (Aspergillus flavus partitivirus 1, Colletotrichum acutatum RNA virus 1, Botryosphaeria dothidea virus 1, Valsa cypri partitivirus and Ustilago virens partitivirus 2 and 3) were found in the proposed genus Zetapartitivirus, an unclassified cluster that groups outside of the five genera in family *Partitiviridae* [38].

The 5′-untranslated regions (UTRs) of PvPV-1 dsRNA1 and dsRNA2 are 14 and 113 bp long, respectively, and show conserved nucleotides at the 5′-termini (AAACTTTTGT) (Fig. 2b). A similar conserved region (AAACTTTTGT) is present in the 5′ UTR of Botryosphaeria dothidea virus 1. The 3′-UTRs differ in length, 94 bp for dsRNA1 and 130 bp for dsRNA2, and show a conserved nucleotide stretch of small and long sequences with a conserved stretch (ATTCCCTCGATTC) at the 3′-terminus (Fig. 2b). This is in accordance with the observations for multi-component RNA viruses, where the 5′-terminal sequences are important for recognition by the RdRP during the replication of viral genome [39]. Possible secondary structures were predicted for the 5′- and 3′- termini of both PvPV-1 dsRNA 1 and dsRNA 2 (Fig. S1, available in the online version of this article). Such structures give stability and strength to the RNA molecule or genome and may have a role in viral replication and assembly [40].

### Construction of isogenic lines and completion of Koch’s postulates

**Fig. 4.**
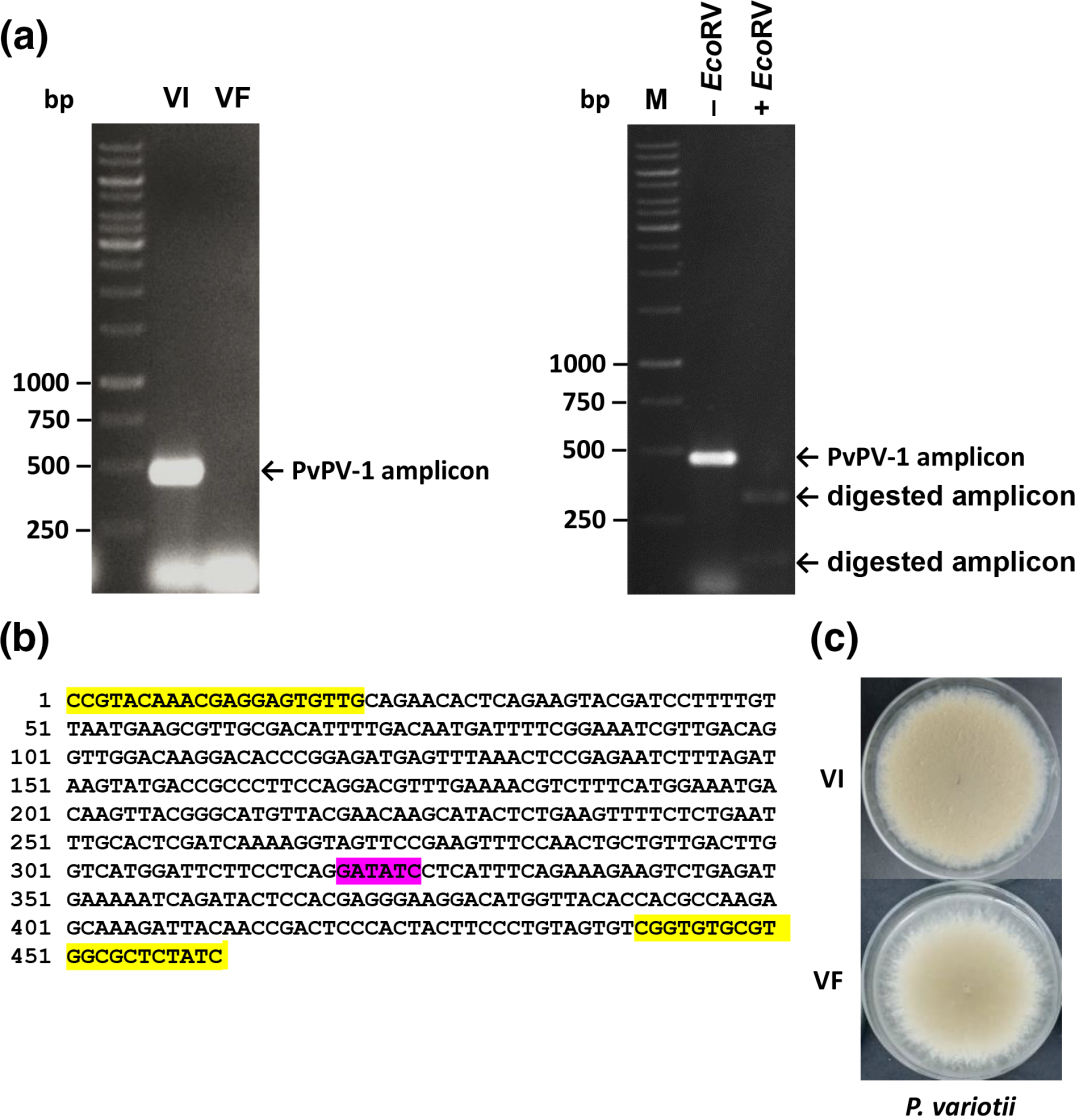
PvPV-1 elimination. (**a**) Agarose gel electrophoresis of the PvPV-1 RdRP amplicon following RT-PCR of *P. variotii* virus-infected (VI) and virus-free (VF) isogenic lines before and after digestion with *EcoRV*. The molecular sizes of the Thermo Fisher Scientific 1 kbp DNA marker are indicated on the left of the gels. (**b**) The PvPV-1 RdRP amplicon sequence (466 bp) showing the forward and reverse primers (highlighted in yellow) and the *EcoRV* restriction enzyme site (highlighted in magenta). (**c**) Colony morphologies of *P. variotii* isogenic lines on PDA.

Cycloheximide at 150 mM concentration successfully cured *P. variotii* from mycoviral infection. The virus-free isogenic line was confirmed by RT-PCR amplification using virus-specific primers. The virus-specific amplicon fractionated by agarose gel electrophoresis was present in the wild-type PvPV-1-infected isolate but absent from the virus-free isogenic line (Fig. 4a). The RT-PCR product was further treated with restriction endonuclease *Eco*RV to confirm the presence of a virus-specific amplicon, generating, as expected, two fragments that were 319 and 147 bp in size (Fig. 4a, b). Colony morphologies of virus-infected and virus-free isogenic lines on PDA are shown in Fig. 4c.

Subsequently, hyphal fusion between virus-infected (donor) and virus-free (recipient) isogenic lines was successfully performed, as shown in Fig. S2a,b, for horizontal transmission of PvPV-1. Selected regions (Fig. S2c) of both donor and recipient mycelia were assessed for mycovirus presence and dsRNA extraction followed by agarose gel electrophoresis confirmed virus transfer from donor to recipient (Fig. S2d). This experiment completes Koch’s postulates, an important step for establishing a cause–effect relationship, and ensures genetically identical isogenic lines, as treatment with chemicals such as cycloheximide is known to cause mutations [41].

### Effects of PvPV-1 infection on *P. variotii* radial growth, biomass, metabolism and antagonistic potential

In order to determine the effects of PvPV-1 infection on *P. variotii*, virus-free and virus-infected isogenic lines were analysed in terms of radial growth on different solid media and biomass production in PDB [42, 43]. These experiments included the original virus-infected (VI) isolate, the cured virus-free (VF) isolate, and one of the recipients of PvPV-1 following hyphal fusion (R5).

**Fig. 5.**
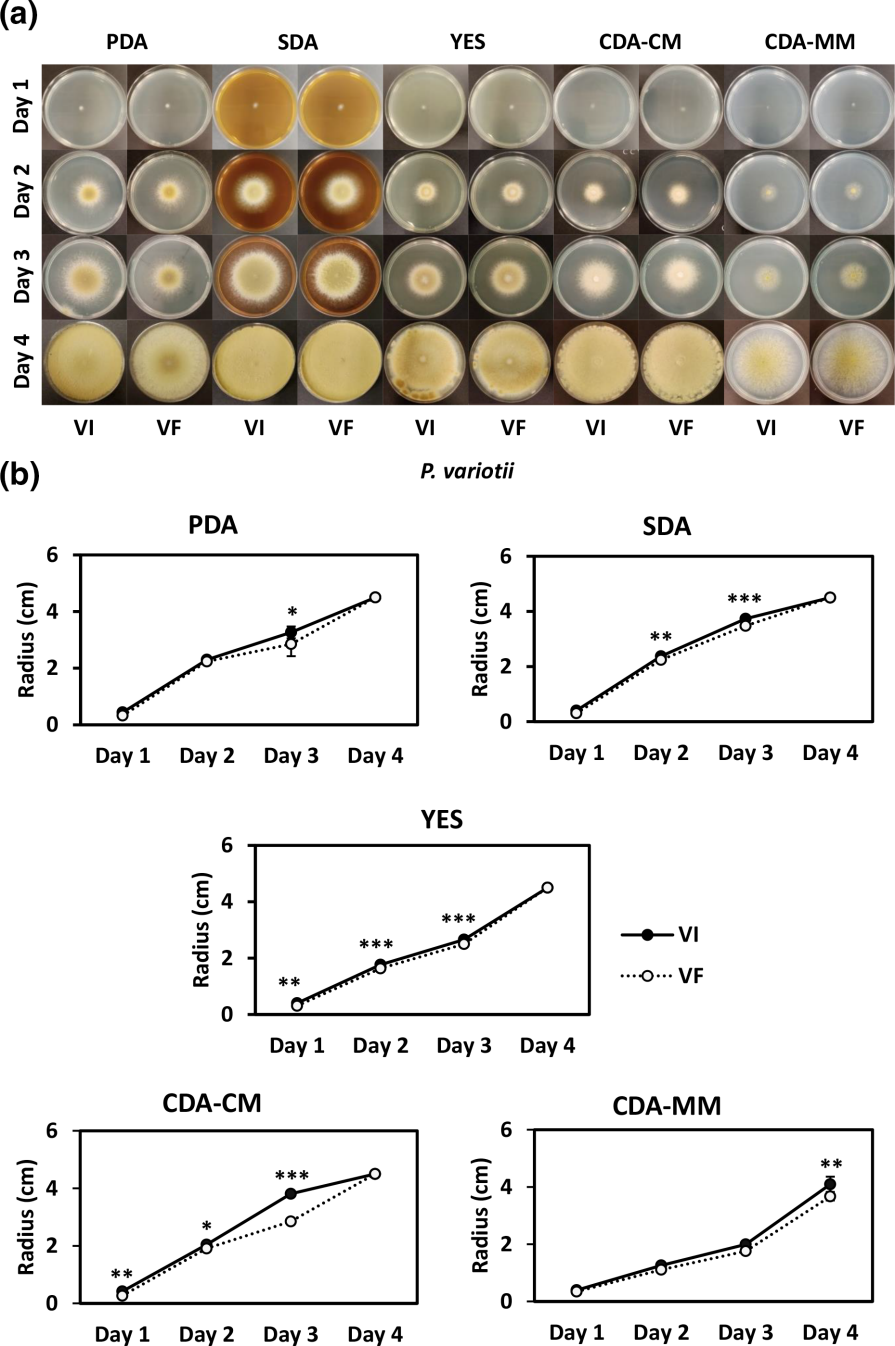
Radial growth of *P. variotii* isogenic lines. (**a**) Colony morphologies and (**b**) radial growth of *P. variotii* virus-infected (VI) and virus-free (VF) isogenic lines on different media, including PDA, SDA, YES, CDA-CM and CDA-MM, over a period of 5 days. Asterisks indicate statistical significance following two-way ANOVA: *, *P*-value<0.05; **, *P*-value <0.01; ***, *P*-value<0.001.

**Fig. 6.**
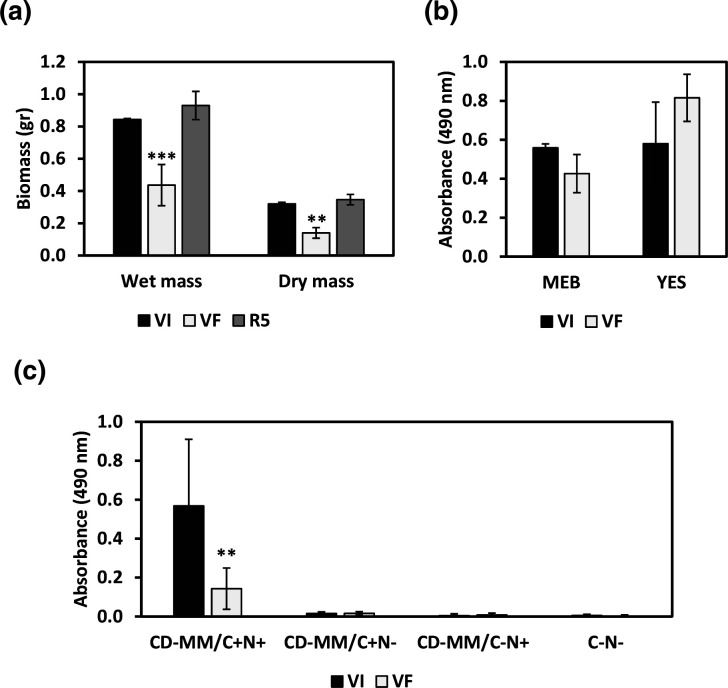
Biomass and metabolism of *P. variotii* isogenic lines. (**a**) Biomass production of *P. variotii* virus-infected (VI), virus-free (VF) and virus recipient (**R5**) isogenic lines in PBD. Asterisks indicate statistical significance following Student’s *t*-test: **, *P*-value <0.01; ***, *P*-value <0.001. (**b**) Metabolic activity of *P. variotii* isogenic lines in MEB and YES. (**c**) Metabolic activity of *P. variotii* isogenic lines in CD-MM with and without carbon and nitrogen sources. Asterisks indicate statistical significance following two-way ANOVA: **, *P*-value <0.01.

The radial expansion of VI, VF and R5 was measured over a period of up to 5 days on different growth media. In all cases, the radial growth of VI and R5 is faster as compared to VF and this observation was confirmed as statistically significant for some time points using two-way ANOVA (*P*-value <0.05). The colony morphologies of VI and VF grown on different media are shown together with the graphical representation of the diameters recorded in Fig. 5a, b respectively. Comparisons of VF and R5 radial expansion were performed in smaller Petri dishes and a graphical representation of the diameters recorded is shown in Fig. S2. Further, both the wet and the dry biomass production of VI and R5 were significantly greater (*P*-value <0.01) as compared to VF, as shown by Student’s *t*-test (Fig. 6a).

Conversely, no statistically significant differences in the metabolic activity of VI and VF *P. variotii* were noted in MEB and YES (Fig. 6b). However, cultures of VI *P. variotii* grown in CD minimal media containing sucrose and sodium nitrate as carbon and nitrogen carbon sources, respectively, were darkly pigmented with formazan (a coloured compound produced because of reduction of XTT by the dehydrogenase enzyme produced by metabolically active cells) [44], whereas VF cultures and those deprived of carbon and nitrogen were lighter in colour with minimum optical density values being recorded by spectrometry, presumably because of limited fungal growth (Fig. 6c). The difference between the metabolic activity of VI and VF *P. variotii* was statistically significant (*P*-value <0.01), as shown using two-way ANOVA.

**Fig. 7.**
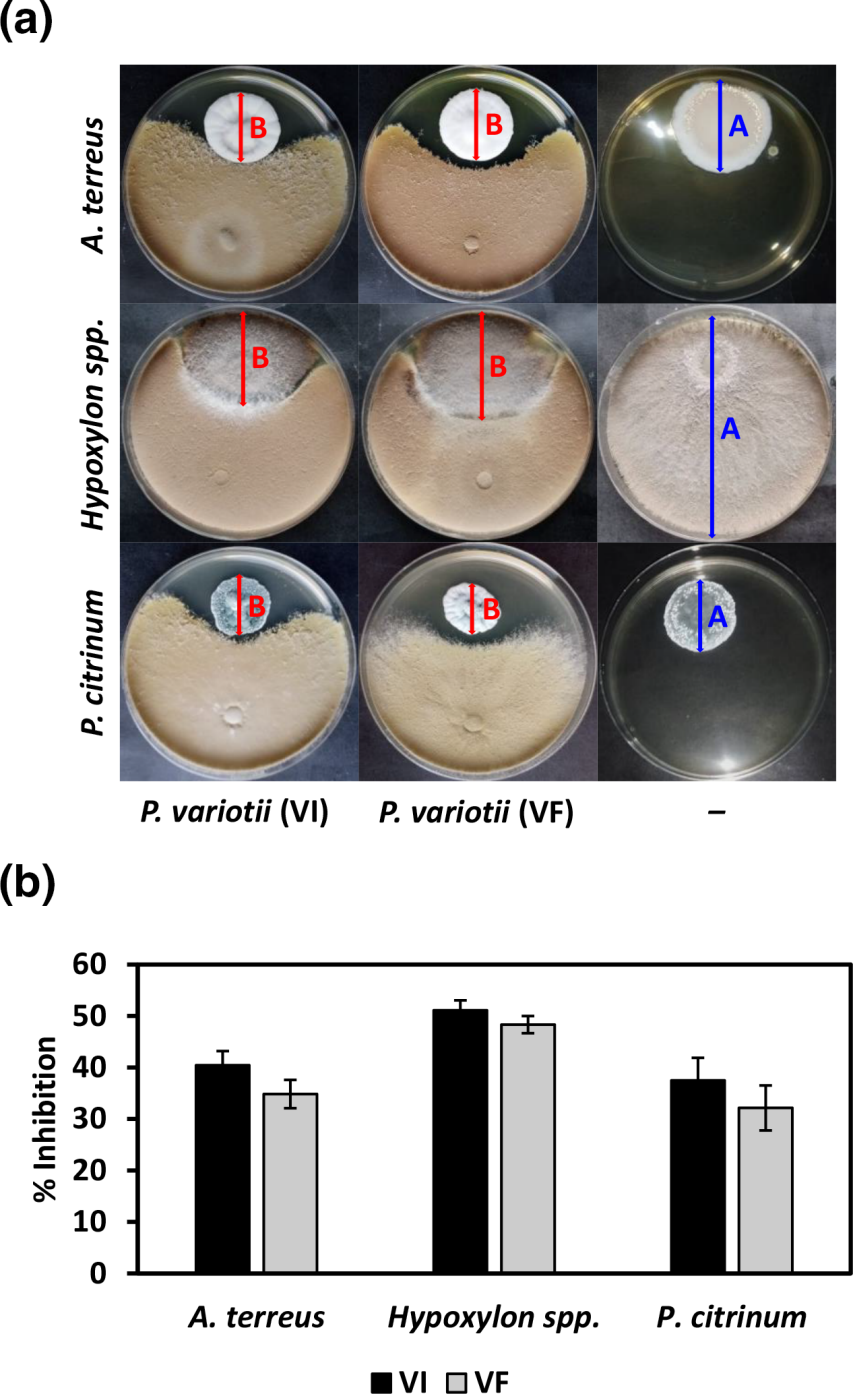
Antagonistic potential of *P. variotii* isogenic lines. (**a**) Dual-culture assay and (**b**) percentage inhibition of *P. variotii* virus-infected (VI) and virus-free (VF) isogenic lines against three plant pathogenic fungi, *A. terreus, Hypoxylon* spp. and *P. citrinum*. The blue and red arrows in (**a**) indicate the colony diameter of the fungal pathogens in control (A) and treated (B) groups, respectively, as measured during exemplar dual-culture assays to calculate percentage inhibition in (b). Two-way ANOVA indicated an overall statistical significance with *P*-value <0.01 when comparing *P. variotii* isogenic lines.

Finally, the antagonistic potential of isogenic lines of *P. variotii* against different fungi, including *A. terreus, Hypoxylon* spp*. and P. citrinum*, was evaluated using a dual-culture assay. The percentage inhibition, ranging from 30 to over 50%, was found to be greater for the VI line as compared to the VF line (Fig. 7a, b). This overall trend was shown to be statistically significant (*P*-value <0.01) when comparing the effect of isogenic lines using two-way ANOVA, although pairwise comparisons did not reveal any significance.

## Discussion

*P. variotii* is a cosmopolitan fungus that has a significant role as a biocontrol agent and in industry as a source of industrial tannase [18, 45]. To date, there have been no reports of mycovirus infection in *P. variotii* and this study is the first to describe the presence of icosahedral VLPs and dsRNA elements from a novel mycovirus, PvPV-1. PvPV-1 has been unambiguously identified as a new member of the family *Partitiviridae* and has all the traditional hallmarks of a partitivirus: non-enveloped icosahedral VLPs [46] that encapsidate two genomic dsRNA segments 1.3–2.5 kbp in size encoding respectively RdRP and CP and show high sequence identity of their 5′-UTRs [39]. The presence of one or more additional genomic components has been reported in several partitiviruses, but there is no such evidence for PvPV-1. PvPV-1 RdRP and CP are both significantly similar in sequence to proteins encoded by Aspergillus flavus partitivirus 1, Aspergillus niger partitivirus 1, Botryosphaeria dothidea virus 1, Colletotrichum acutatum RNA virus 1 and Valsa cypri partitivirus (Table S1). Phylogenetic analysis indicates that this group of mycoviruses clusters in an unclassified taxon unrelated to the existing five genera within *Partitiviridae* (Fig. 3). Based on our findings, PvPV-1, together with related viruses, represents the new genus named Zetapartitivirus [8] according to the *Partitiviridae* nomenclature. The species demarcation criteria within each genus of the family *Partitiviridae* are ≤90 and ≤80 % aa sequence identity in respectively the RdRP and the CP; therefore, PvPV-1 represents a new species within Zetapartitivirus.

Viruses in the family *Partitiviridae* are associated with persistent infections of their fungal, plant, insect and protozoan hosts [47, 48], do not generally elicit any obvious symptoms in their hosts and are considered to be latent [39, 49–51]. However, some partitiviruses confer hypovirulence to their plant-pathogenic hosts, including Sclerotinia sclerotiorum partitivirus 1 [52], Rosellinia necatrix partitivirus (RnPV) 10,11, 14, 15, 16 and 20 [53], Rhizoctonia solani partitivirus 2 [54] and Heterobasidion partitivirus 13 [55]. Other examples of non-latent partitiviruses include Aspergillus fumigatus partitivirus 1, which alters colony morphology, reduces pigmentation and slows growth [56], and Flammulina velutipes browning virus, which is associated with the cap colour of fruiting bodies [52, 57–59].

Investigating mycovirus infection in fungi used as biocontrol agents is important, as their antagonistic potential may vary, depending on mycovirus-mediated phenotypes, including effects on host growth. For instance, mycovirus-mediated increased host growth may be advantageous, since it could restrict growth of other phytopathogenic fungi, reducing pathogenicity and controlling disease. Growth inhibition of pathogenic fungi by a biocontrol agent, such as mycovirus-infected *Trichoderma* spp., was previously assessed to investigate potential differences in percentage inhibition. Trichoderma harzianum partitivirus 1 (ThPV-1) infection led to no significant differences in colony morphology or pigmentation of the host *Trichoderma harzianum*. However, growth inhibition of *Pleurotus ostreatus* and *Rhizoctonia solani* by *T. harzianum* was increased in the VI strain as compared with the VF isogenic strain in dual-culture experiments. Moreover, β−1,3-glucanase but not chitinase activity was significantly increased in the VI strain, suggesting that ThPV-1 has a role in regulating the activity of a specific host enzyme [51]. A study on two *Trichoderma* spp. strains infected with other mycoviruses beyond *Partitiviridae* reported that mycovirus infection decreased their growth rate, sporulation and biocontrol efficacy [60]. Finally, dsRNA elements in *T. harzianum* were reported to decrease its biocontrol potential but improved plant growth [61].

In this study, PvPV-1 infection increased *P. variotii* growth based on comparisons of VF and VI isogenic lines in radial expansion assays and biomass measurements in liquid media. This observation is at least partially explained through the enhanced metabolic activity of VI *P. variotii*, as illustrated by XTT metabolic assays. Additionally, PvPV-1 improved the antagonistic potential of *P. variotii* by increasing its percentage growth inhibition of pathogenic fungi. This observation may stem from increased growth of the VI isolate as compared to the VF isolate. Our results are in accordance with previous studies illustrating the antagonistic potential of *P. variotii* [18, 62, 63]. In conclusion, we isolated and characterized a novel partitivirus that confers interesting phenotypes on its host and to our knowledge this represents the first report of mycovirus infection in *P. variotii*.

## Supplementary Data

Supplementary material 1
