# Supplementary material for: Molecular and biological characterization of a partitivirus from Paecilomyces variotii
Source: J Gen Virol. 2023 Nov 28;104(11):001925. doi: 10.1099/jgv.0.001925 (PMC10768695; doi:10.1099/jgv.0.001925)
Supplement: Supplementary material 1 [file jgv-104-1925-s001.pdf]

Table S1. PvPV-1 encoded proteins and their homologues as revealed by BLASTp.

Virus	dsRNA1 RdRP			dsRNA2 CP		
	Identity (%)	E-value	Accession	Identity (%)	E-value	Accession
Aspergillus flavus partitivirus 1						
	82.44	0.0	QDE53634	57.03	2e-147	QDE53635
Aspergillus niger partitivirus 1						
	79.85	0.0	BDF97658	58.33	1e-146	BDF97659
Botryosphaeria dothidea virus 1						
	82.81	0.0	AIE47694	60.27	9e-157	AIE47695
Colletotrichum acutatum RNA virus 1						
	77.74	0.0	AGL42312	57.22	6e-146	AGL42313
Valsa cypri partitivirus						
	84.09	0.0	AIS37548	55.93	7e-78	AIS37549

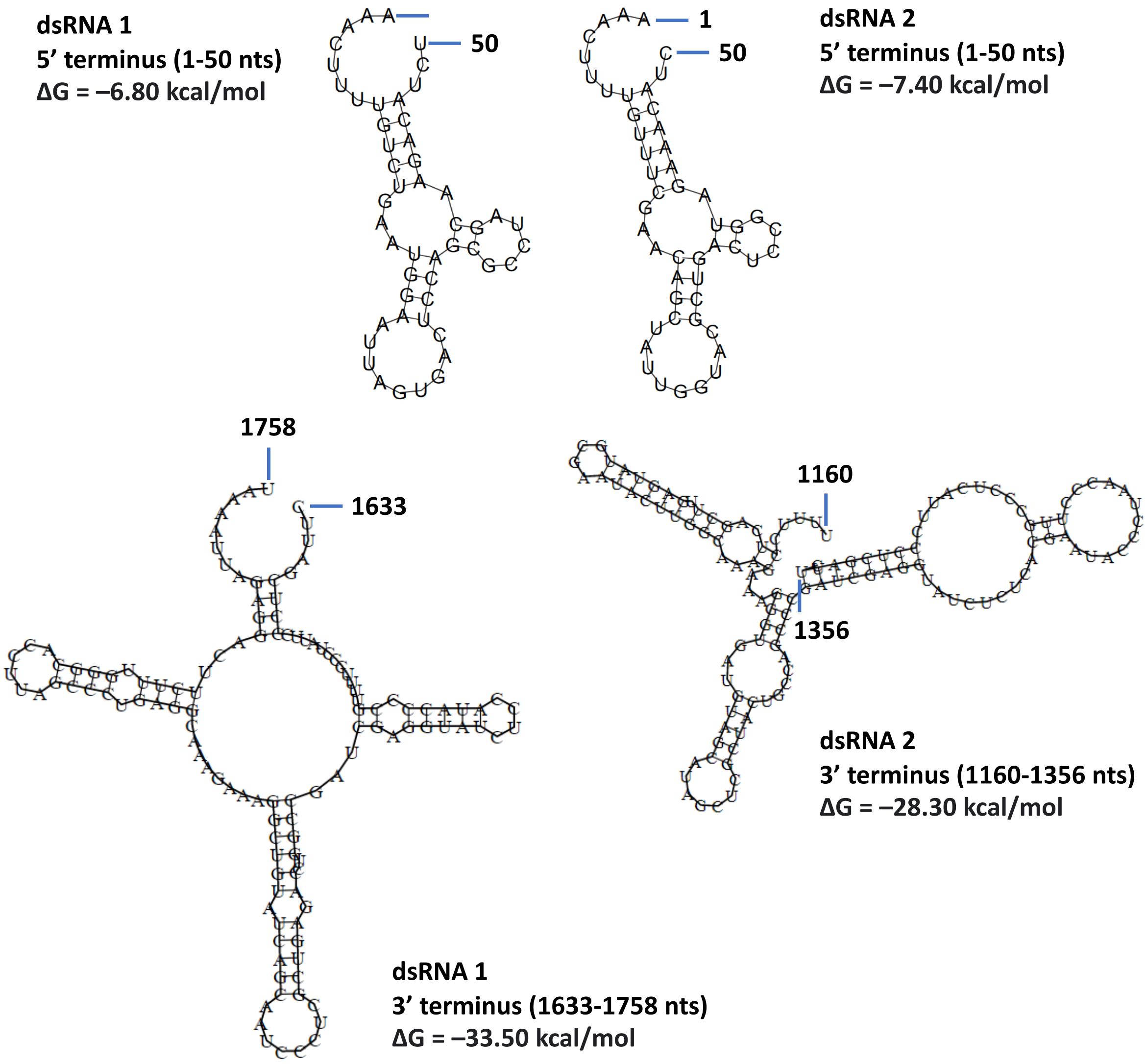

Figure S1. Predicted secondary structures of the 5' and 3' UTRs of PvPV-1 dsRNA1 and dsRNA2 using online RNAfold 2.5.1. The minimum free energy (ΔG) is shown with each predicted structure.

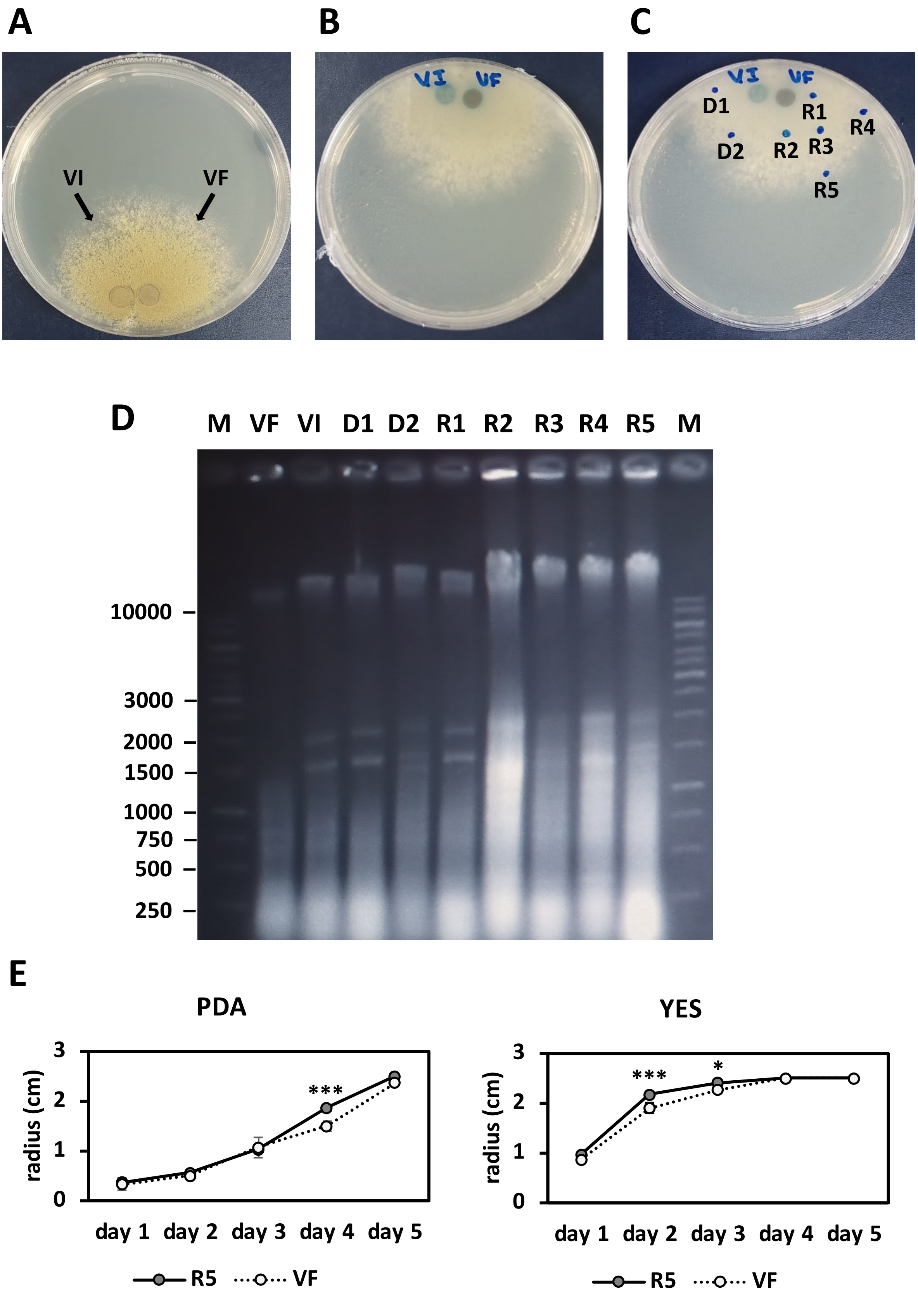

Figure S2. PvPV-1 horizontal transfer. (A) Front view and **(B)** rear view of hyphal fusion between isogenic virus-infected (VI) and virus-free (VF; following cycloheximide treatment) *P. variotii* isolates. **(C)** Selected regions of donor and recipient subcultured and assessed for

PvPV-1 presence. **(D)** Agarose gel electrophoresis of extracted dsRNA extracted from negative control (VF), positive control (VI), donor (D1-2) and recipient (R1-5). The molecular sizes of Thermo Fisher Scientific 1 kbp DNA marker are indicated on the left of the gels. **(E)** Radial growth of *P. variotii* virus-free (VF) and virus recipient (R5) isogenic lines on different media, including PDA and YES over a period of 5 days.
